# Extracellular vesicles: a missing component in plant cell wall remodeling

**DOI:** 10.1093/jxb/ery255

**Published:** 2018-07-11

**Authors:** Laura de la Canal, Marcela Pinedo

**Affiliations:** Instituto de Investigaciones Biológicas, Universidad Nacional de Mar del Plata – CONICET, Funes, Mar del Plata, Argentina

**Keywords:** Cell wall, exosomes, extracellular vesicles, glycoside hydrolases, leaderless proteins, unconventional protein secretion (UPS)

**In animal systems extracellular vesicles (EV) are known to transport cargo molecules from the cytoplasm to the extracellular compartment and they are the accepted vehicles for unconventional protein secretion. Plants have recently been shown to release EV into the apoplast and here we postulate a role in cell wall remodeling. Delving deeper into our proteomics data we found that much of the protein complement of EV from sunflower seedlings corresponds to cell wall-related proteins, including enzymes that participate in the degradation and reorganization of polysaccharides. Accumulated data implicate EV in the unconventional secretion of cell wall-modifying enzymes.**

Cells can release various types of nano-sized membrane vesicles into their environment, called extracellular vesicles (EV), and these are known to transport cargo molecules to the extracellular fluids and between cells as a form of intercellular communication (Maas *et al.*, 2017). The generic term EV includes a range of vesicles of different size and cellular origin, including exosomes, microvesicles and apoptotic bodies (Yáñez-Mó *et al.*, 2015). Even though most of our knowledge on EV comes from mammalian systems, it is accepted that virtually all living cells including archaea, bacteria, and eukaryotes secrete nano-vesicles into the extracellular space. Research in plant systems is just beginning, and two recent reports have analyzed the proteome of EV obtained from apoplastic fluids of pathogen-infected and uninfected Arabidopsis rosettes (Rutter and Innes, 2017) and sunflower seedlings not subjected to biological stress (Regente *et al.*, 2017). Defense proteins were enriched in both cases and the involvement of EV in plant defense was suggested. Sunflower EV were also shown to be taken up by the spores of a fungal pathogen leading to growth restriction and cell death (Regente *et al.*, 2017). Accordingly, a role for EV in plant–pathogen interactions is emerging (Boevink, 2017; Hansen and Nielsen, 2018) and they have been considered key mediators of such interactions (Rutter and Innes, 2018). Thus, current knowledge indicates that plant EV could participate in intercellular communication as previously described in mammals. In addition to the EV enrichment in defense proteins analyzed in these papers, cell wall-related proteins were detected in both experimental systems. Some of the common families found in sunflower and Arabidopsis EV include glycosyl hydrolases, fasciclin-like arabinogalactan proteins, lectins, leucine-rich-repeat proteins and lipase acylhydrolases (Regente *et al.*, 2017; see Supplementary Table S9). Since the cell wall plays central roles in plant physiology we decided to investigate EV as putative carriers of proteins involved in cell wall assembly/modification in the extracellular compartment.

## Protein secretion for cell wall building and modification

Plant cells are characterized by the presence of a surrounding wall that confers mechanical strength and plays a key role in controlling their size and shape. The cell wall has a highly dynamic composition and architecture which differs according to cell types as well as developmental, growth and environmental conditions. Its complex structure is synthesized and maintained by a large number of proteins. To accomplish its functions, each cell wall requires a precise carbohydrate and protein composition which depends on transport pathways from the cytosol to the apoplast (Keegstra, 2010; Lampugnani *et al.*, 2018). In fact, it is accepted that a central role of protein secretion in plant cells is to allow cell wall building and remodeling (Kim and Brandizzi, 2014; van de Meene *et al.*, 2017). The classical view of secretion of enzymes involved in cell wall polysaccharide synthesis and modification assumes that they are synthesized and delivered using the conventional secretory pathway. This means that those enzymes exhibit an N-terminal leader sequence which drives them to the endoplasmic reticulum (ER) to continue their synthesis and modification through the endomembrane system. They move to the Golgi apparatus and then, using secretory vesicles, are released into the extracellular compartment or directed to the plasma membrane. However, this is not the only possible route of protein secretion; alternative pathways have been described and these are an active field of investigation in animal and yeast models (Rabouille, 2017). In plants, their contribution to the cell wall proteome has been analyzed (Rose and Lee, 2010) and, even if the transport mechanisms for cell wall enzymes are not fully understood, evidence for unconventional secretion of a few of those proteins has been obtained (reviewed in Davis *et al.*, 2016).

**Fig. 1.**
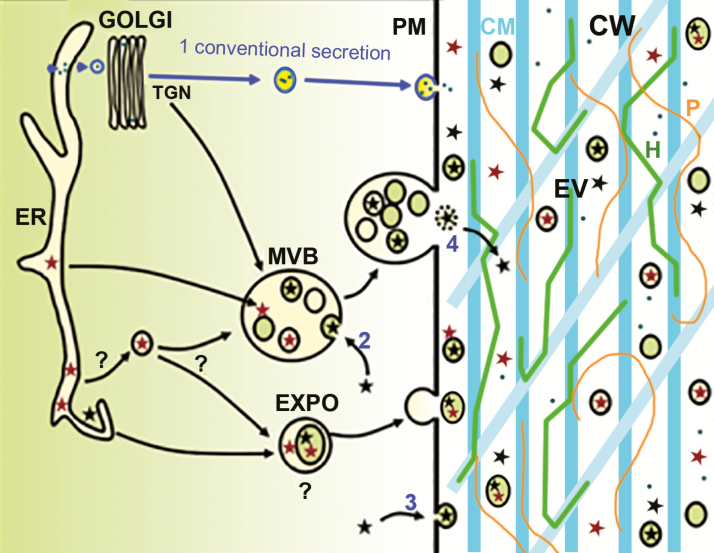
Secretion of extracellular vesicles carrying cell wall-remodeling proteins. The diagram shows conventional and unconventional protein secretion. In conventional secretion of proteins with signaling peptides, these enter the endoplasmic reticulum (ER) before passing through the Golgi, *trans*-Golgi network (TGN) and vesicles that fuse with the plasma membrane (PM) (1). In unconventional secretion of proteins, those that do have signaling peptides (red stars) and leaderless proteins (black stars) move via Extracellular Vesicles (EV) originating from Multivesicular Bodies (MVB) (2), budding from the PM (3) or exocyst positive organelles (EXPO) followed or not by degradation of the vesicle membrane in the apoplast (4). Connections between the ER and MVB or EXPO are proposed (question marks). Cell wall (CW) components are indicated: CM, Cellulose Microfibrils; H, Hemicellulose; P, Pectin. This diagram is adapted from Boevink (2017).

Putative mechanisms for unconventional protein secretion (UPS) in plants have been proposed based on some experimental data and the demonstrated routes in other *eukaryotes* (Goring and Di Sansebastiano, 2018). Some of the proposed pathways include intracellular vesicular carriers such as multivesicular bodies (MVB) and *exocyst*-positive organelles (EXPO). MVB may fuse to the plasma membrane to liberate exosome-like vesicles in the extracellular matrix and the EXPO has also been proposed to release extracellular vesicles into the apoplast. Thus, both exosomes and EXPO-derived vesicles have been suggested as agents for UPS in plants (Wang *et al.*, 2010). Kim and Brandizzi (2014) highlighted a putative role of EXPO for cell wall building. Additionally, microscopic evidence shows that at least some EV might be retained in the cell wall to deliver materials required for *cell wall synthesis* (An *et al.*, 2006; Wang *et al.*, 2010). Nevertheless, to our knowledge, no direct evidence for EV in cell wall processes has been presented. Therefore, a model involving EV is proposed and investigated here (Fig. 1). In this context, we decided to analyze proteomics data to evaluate the potential contribution of EV to the secretion of cell wall-related proteins into the extracellular compartment.

## Cell wall-related proteins in sunflower EV

Previous proteomics data obtained from an EV-enriched fraction isolated from the apoplast of *Helianthus annuus* seedlings by ultracentrifugation (Regente *et al.*, 2017; see Supplementary Table S7) were clustered according to their gene ontologies (GO) using AgriGO, a toolkit designed to perform ontology enrichment analysis on agricultural species (Du *et al.*, 2010). Analysis of the biological processes associated with those proteins revealed the enrichment of several GO related to polysaccharide metabolism when the list of genes corresponding to the identified proteins was compared to whole-genome clustering (see Supplementary Fig. S1 at *JXB* online). ‘Cell wall macromolecule catabolic process’ (GO 0016998) was significantly over-represented (4 × 10^7^) together with the related ‘carbohydrate metabolic process’ (GO 0005975) and ‘polysaccharide catabolic process’ (GO 0000272) (Supplementary Fig. S1). Those proteomics data were then used to examine cell wall-related proteins in the nine functional classes defined by Jamet *et al.* (2008). Notably, 112 proteins out of 237 were clustered as cell wall proteins (Supplementary Table S1). They comprise many proteins implicated in cell wall reorganization in other species (Houston *et al.*, 2016) such as glycosyl hydrolases (GH), expansins and arabinogalactan proteins. Other cell wall proteins detected are proteases, Kunitz-inhibitors, peroxidases, GDSL lipases, germin-like proteins, lipid-transfer proteins and multicopper oxidases. Briefly, proteins belonging to eight out of the nine functional classes defined for Arabidopsis cell wall proteins were found in the sunflower EV fraction. They are proteins acting on carbohydrates, oxido-reductases, proteases, proteins with interaction domains, proteins possibly involved in signaling, proteins related to lipid metabolism, miscellaneous proteins (mainly germins) and proteins with unknown function (DUF). On the other hand, no proteins belonging to the class ‘structural proteins’ were detected. GH appeared well represented in the ultracentrifugation pellet, with 35 different proteins belonging to 15 families out of more than 120 classified in the CAZy database. Several GH families found associated to EV (such as GH 3, 16, 27, 31 and 35) were previously described in cell wall proteomes (Jamet *et al.*, 2008) and have been involved in the reorganization of cell wall carbohydrates during cell growth (Minic and Jouanin, 2006).

Overall, 47% of the proteins identified in the EV-enriched fraction from sunflower apoplastic fluids are predicted to be cell wall-related proteins. Since the protein fraction analyzed was isolated by a 100 000 *g* centrifugation without further vesicle purification the putative presence of cell wall aggregates cannot be completely excluded. Nevertheless, we failed to detect pectin-derived uronic acids in this fraction. It must be emphasized that cell wall-related proteins were also detected in Arabidopsis EV purified using a density gradient (Rutter and Innes, 2017).

Together with the gene ontology analysis, this clustering suggests that EV do seem to be central vehicles for the secretion of cell wall proteins in sunflower seedlings under basal conditions, since such proteins were found in an EV fraction obtained from plants not subjected to biotic stress. Cell wall-remodeling enzymes appeared to be associated with EV and therefore suggest a role for EV in the modification of cell wall polysaccharides during plant growth and development. To date only a role for EV in plant defense against pathogens has been proposed, but it is known that cell wall composition is related to plant immunity (Underwood, 2012; Bethke *et al.*, 2016). Thus part of the defense function of EV may be related to their participation in cell wall remodeling. For example, Arabidopsis PMR5 is a cell wall protein involved in pectin methyl esterification which appeared to have a role in penetration resistance against fungal pathogens (Vogel *et al.*, 2004; Engelsdorf *et al.*, 2017). Intriguingly, a PMR5 homolog is one of the cell wall proteins detected here associated with EV (see Supplementary Table S1 at *JXB* online).

As a next step, we examined whether the EV pellet fraction was enriched in leaderless cell wall proteins that might follow non-classical secretion. We recovered data regarding the presence/absence of an N-terminal signaling peptide from previous analyses (Regente *et al.*, 2017; see Supplementary Table S7). Supplementary Table 1 shows that most of the cell wall-related proteins detected are predicted to have an N-signaling peptide in their coding sequence. In fact, 100 out of 112 proteins are predicted to follow a classical secretion pathway based on the occurrence of that signal. On the other hand, a minor part (12 proteins) was classified as ‘non-secretory’ protein based on the absence of the N-signaling peptide and of pre-sequences for targeting to chloroplasts and mitochondria. These leaderless proteins include the lectin Helja (HanXRQChr02g0047121) whose non-classical secretion has been experimentally demonstrated (Pinedo *et al.*, 2012). Interestingly, the leaderless serine carboxypeptidase detected in sunflower EV (HanXRQChr03g0072241) is a member of the peptidase family S10, the same family as CPY, another known plant protein following UPS (Hatsugai *et al.*, 2009; Davis *et al.*, 2016).

The prevalence of cell wall proteins carrying a signaling peptide in EV may appear unexpected since absence of the N-terminal signal is generally considered a feature of a protein following UPS (Nickel, 2003). Nevertheless, this concept has been revised and, in addition to the ER–Golgi-independent pathway, models for UPS in animals propose other trafficking possibilities such as a bypass of the Golgi after entering the ER in a signaling peptide-dependent manner (Rabouille, 2017). The biogenesis of EV in plants has not yet been characterized but it is striking that the two EV proteomes currently available display proteins with and without signaling peptides. The high proportion of proteins with predicted signaling peptides in plant EV may reflect their unknown origin and transport mechanisms which require further studies to be understood.

Regardless of the mechanism used by plant cells to produce EV, their enrichment in cell wall proteins highlights their contribution to the secretion of cell wall components. Even if our knowledge of plant EV is still at a preliminary stage, our results suggest a role of these vesicles in modifying the composition of plant cell walls according to the diagram in Fig. 1. Briefly, cell wall-related proteins can follow a classical secretory pathway to reach the apoplast after fusion of secretory vesicles with the plasma membrane. UPS pathways can also liberate proteins into the extracellular compartment using different mechanisms, some of them involving EV. Although EV biogenesis in plants is still unknown, proposed pathways include EXPO fusion or MVB fusion to the plasmalemma (Wang *et al.*, 2017). These mechanisms would be able to release vesicles into the apoplast that can directly interact with cell wall components or, alternatively, plant EV could also burst or break in the extracellular compartment to release their contents and exert their activity. These proposed pathways are consistent with experimental data demonstrating that some cell wall-related proteins follow a UPS mechanism probably involving EV derived from MVB or EXPO (Davis *et al.*, 2016). This is the case for an arabinogalactan glycosyltransferase (Poulsen *et al.*, 2014), the *S*-adenosylmethionine synthase 2 (Wang *et al.*, 2010) and the UDP-glucuronate epimerases 1 and 6 (Poulsen *et al.*, 2015). Our results suggest that these examples may not be exceptional cases but rather the first among a large number of cell wall proteins secreted using an EV-dependent mechanism. EV may then be part of the toolkit used by plants to ensure a dynamic remodeling of the cell wall structure able to adapt to changing environmental and growing conditions.

## Conclusions

EV research in mammals has mainly focused on their role in intercellular communication. Our data suggest that plant EV may also have a role in the maintenance of cell structure and function through the secretion of cell wall-related proteins. Cell wall-modifying enzymes appeared associated with EV, revealing an unexplored contribution of these vesicles in the reorganization of the cell wall, a crucial process for plant growth, development and adaptation to environmental conditions. Future studies regarding routes for non-classical protein secretion through EV should clarify their biogenesis and potential cargo selection of cell wall components.

## Supplementary data

Supplementary data are available at *JXB* online.

Table S1. Cell wall proteins identified in the sunflower EV pellet fraction (Badouin *et al.*, 2017).

Fig. S1. Gene ontology enrichment analysis of proteins identified in sunflower EV.

## Supplementary Material

Supplementary Table S1

Supplementary Figure S1
