# Supplementary material for: Extracellular vesicles: a missing component in plant cell wall remodeling
Source: J Exp Bot. 2018 Jul 11;69(20):4655–8. doi: 10.1093/jxb/ery255 (PMC6137967; doi:10.1093/jxb/ery255)
Supplement: Supplementary Table S1 [file ery255_suppl_supplementary_table_s1.doc]

## Supplementary Table 1: Cell wall proteins identified in sunflower EV pellet fraction

Data were extracted from the total proteome unambiguously identified in the EV fraction isolated from sunflower apoplast by ultracentrifugation (237 proteins) and presented in Supplementary Table S7 from Regente et al. (2017). Cell wall proteins were clustered in the classes described by Jamet *et al.* (2008) according to their functional annotation. Accession corresponds to the sunflower database (<https://www.heliagene.org/HanXRQ-SUNRISE/>) (Badouin *et al.*, 2017). TargetP/Signal P prediction indicates the presence of N-terminal signal peptide (+) or the absence of signal peptide and prediction as Non-secretory protein (-).

| Accession | **Functional annotation** | **TargetP/Signal P**  **prediction** |
| --- | --- | --- |
|  |  |  |
| **With predicted N-terminal**  **signal peptide (100)** | |  |
|  |  |  |
| HanXRQChr05g0142271 | aldose-1-epimerase | + |
| HanXRQChr09g0247561 | Asp protease (Peptidase family A1) | + |
| HanXRQChr03g0063871 | Asp protease (Peptidase family A1) | + |
| HanXRQChr03g0080191 | Asp protease (Peptidase family A1) | + |
| HanXRQChr09g0248241 | Asp protease (Peptidase family A1) | + |
| HanXRQChr14g0452111 | Asp protease (Peptidase family A1) | + |
| HanXRQChr06g0184671 | Asp protease (Peptidase family A1) | + |
| HanXRQChr15g0481451 | Asp protease (Peptidase family A1) | + |
| HanXRQChr14g0431181 | Asp protease (Peptidase family A1) | + |
| HanXRQChr15g0484151 | Asp protease (Peptidase family A1) | + |
| HanXRQChr13g0409681 | berberine-bridge enzyme (S)-reticulin:oxygen oxido-reductase | + |
| HanXRQChr11g0334771 | carbohydrate esterase family 13 - CE13 (pectin acylesterase) | + |
| HanXRQChr12g0360101 | carbohydrate esterase family 8 - CE8 (pectin methylesterase) | + |
| HanXRQChr04g0103281 | Cys protease (papain family) | + |
| HanXRQChr09g0267121 | Cys protease (papain family) | + |
| HanXRQChr16g0508901 | Cys protease (papain family) | + |
| HanXRQChr03g0065901 | expansin | + |
| HanXRQChr14g0460101 | expansin | + |
| HanXRQChr09g0244331 | expansin | + |
| HanXRQChr17g0559441 | expansin | + |
| HanXRQChr01g0015821 | expressed protein (DUF642) | + |
| HanXRQChr13g0402501 | expressed protein (DUF642) | + |
| HanXRQChr03g0068311 | expressed protein (LRR domains) | + |
| HanXRQChr10g0291371 | expressed protein (LRR domains) | + |
| HanXRQChr03g0075061 | fasciclin-like arabinogalactan protein (FLA) | + |
| HanXRQChr08g0233371 | fasciclin-like arabinogalactan protein (FLA) | + |
| HanXRQChr06g0181801 | Germin | + |
| HanXRQChr06g0181881 | Germin | + |
| HanXRQChr08g0214281 | Germin | + |
| HanXRQChr12g0360281 | Germin | + |
| HanXRQChr12g0360361 | Germin | + |
| HanXRQChr10g0319691 | Germin | + |
| HanXRQChr10g0312621 | Germin | + |
| HanXRQChr15g0475521 | glycoside hydrolase family 16 - GH16 (endoxyloglucan transferase) | + |
| HanXRQChr12g0376681 | glycoside hydrolase family 16 - GH16 (endoxyloglucan transferase) | + |
| HanXRQChr17g0543231 | glycoside hydrolase family 16 - GH16 (endoxyloglucan transferase) | + |
| HanXRQChr11g0351571 | glycoside hydrolase family 17 - GH17 (beta-1,3-glucosidase) | + |
| HanXRQChr11g0351581 | glycoside hydrolase family 17 - GH17 (beta-1,3-glucosidase) | + |
| HanXRQChr08g0220731 | glycoside hydrolase family 17 - GH17 (beta-1,3-glucosidase) | + |
| HanXRQChr05g0141461 | glycoside hydrolase family 18 - GH18 | + |
| HanXRQChr09g0276721 | glycoside hydrolase family 19 - GH19 | + |
| HanXRQChr09g0276701 | glycoside hydrolase family 19 - GH19 | + |
| HanXRQChr10g0309931 | glycoside hydrolase family 19 - GH19 | + |
| HanXRQChr10g0303711 | glycoside hydrolase family 19 - GH19 | + |
| HanXRQChr10g0303701 | glycoside hydrolase family 19 - GH19 | + |
| HanXRQChr03g0063761 | glycoside hydrolase family 19 - GH19 | + |
| HanXRQChr16g0524981 | glycoside hydrolase family 20 - GH20 (N-acetyl-beta-glucosaminidase) | + |
| HanXRQChr11g0322851 | glycoside hydrolase family 27 - GH27 (alpha-galactosidase/melibiase) | + |
| HanXRQChr08g0208381 | glycoside hydrolase family 27 - GH27 (alpha-galactosidase/melibiase) | + |
| HanXRQChr05g0147191 | glycoside hydrolase family 3 - GH3 | + |
| HanXRQChr01g0026351 | glycoside hydrolase family 3 - GH3 | + |
| HanXRQChr13g0391131 | glycoside hydrolase family 3 - GH3 | + |
| HanXRQChr16g0511881 | glycoside hydrolase family 31 - GH31 (alpha-xylosidase) | + |
| HanXRQChr02g0053981 | glycoside hydrolase family 31 - GH31 (alpha-xylosidase) | + |
| HanXRQChr13g0423671 | glycoside hydrolase family 35 - GH35 (beta-galactosidase) | + |
| HanXRQChr12g0354861 | glycoside hydrolase family 35 - GH35 (beta-galactosidase) | + |
| HanXRQChr02g0055691 | glycoside hydrolase family 35 - GH35 (beta-galactosidase) | + |
| HanXRQChr10g0319301 | glycoside hydrolase family 35 - GH35 (beta-galactosidase) | + |
| HanXRQChr09g0256531 | glycoside hydrolase family 38 - GH38 (alpha-mannosidase) | + |
| HanXRQChr16g0498931 | glycoside hydrolase family 38 - GH38 (alpha-mannosidase) | + |
| HanXRQChr09g0273861 | glycoside hydrolase family 38 - GH38 (alpha-mannosidase) | + |
| HanXRQChr11g0320901 | glycoside hydrolase family 5 - GH5 (glucan-1,3-beta glucosidase) | + |
| HanXRQChr05g0130491 | glycoside hydrolase family 51 - GH51 (alpha-arabinofuranosidase) | + |
| HanXRQChr10g0314191 | glycoside hydrolase family 79 - GH79 (endo-beta-glucuronidase/heparanase) | + |
| HanXRQChr17g0538161 | inhibitor family I3 (Kunitz-P family) | + |
| HanXRQChr05g0158931 | inhibitor family I3 (Kunitz-P family) | + |
| HanXRQChr05g0158941 | inhibitor family I3 (Kunitz-P family) | + |
| HanXRQChr14g0444671 | inhibitor family I3 (Kunitz-P family) | + |
| HanXRQChr14g0444681 | inhibitor family I3 (Kunitz-P family) | + |
| HanXRQChr17g0564111 | lectin (D-mannose) | + |
| HanXRQChr17g0558851 | lectin (D-mannose) | + |
| HanXRQChr06g0170151 | lipase acylhydrolase (GDSL family) | + |
| HanXRQChr10g0287241 | lipase acylhydrolase (GDSL family) | + |
| HanXRQChr11g0343881 | lipase acylhydrolase (GDSL family) | + |
| HanXRQChr16g0504391 | lipase acylhydrolase (GDSL family) | + |
| HanXRQChr16g0504401 | lipase acylhydrolase (GDSL family) | + |
| HanXRQChr09g0258741 | lipase acylhydrolase (GDSL family) | + |
| HanXRQChr01g0000161 | lipid transfer protein/trypsin-alpha amylase inhibitor | + |
| HanXRQChr06g0180691 | lipid transfer protein/trypsin-alpha amylase inhibitor | + |
| HanXRQChr17g0549851 | lipid transfer protein/trypsin-alpha amylase inhibitor | + |
| HanXRQChr05g0143811 | lipid transfer protein/trypsin-alpha amylase inhibitor | + |
| HanXRQChr13g0403071 | lipid transfer protein/trypsin-alpha amylase inhibitor | + |
| HanXRQChr15g0489551 | multicopper oxidase | + |
| HanXRQChr04g0127131 | multicopper oxidase | + |
| HanXRQChr05g0146351 | multicopper oxidase | + |
| HanXRQChr03g0076571 | multicopper oxidase | + |
| HanXRQChr07g0196801 | peroxidase | + |
| HanXRQChr17g0561161 | peroxidase | + |
| HanXRQChr01g0001191 | peroxidase | + |
| HanXRQChr04g0105061 | peroxidase | + |
| HanXRQChr15g0474421 | peroxidase | + |
| HanXRQChr11g0333171 | phosphate-induced (phi) protein 1 | + |
| HanXRQChr11g0328551 | plastocyanin (blue copper binding protein) | + |
| HanXRQChr12g0356851 | purple acid phosphatase | + |
| HanXRQChr02g0055281 | Ser carboxypeptidase (Peptidase family S10) | + |
| HanXRQChr02g0055621 | Ser carboxypeptidase (Peptidase family S10) | + |
| HanXRQChr05g0161101 | Ser carboxypeptidase (Peptidase family S10) | + |
| HanXRQChr13g0387611 | Ser carboxypeptidase (Peptidase family S10) | + |
| HanXRQChr07g0201581 | Ser protease (subtilisin) (Peptidase family S8) | + |
| HanXRQChr12g0377221 | Ser protease (subtilisin) (Peptidase family S8) | + |
|  |  |  |
|  |  |  |
| **Without predicted N-terminal signal peptide (12)** |  |  |
| HanXRQChr09g0251071 | alpha-L-fucosidase | - |
| HanXRQChr06g0175391 | glycoside hydrolase family 16 - GH16 (endoxyloglucan transferase) | - |
| HanXRQChr02g0046191 | glycoside hydrolase family 2 - GH2 | - |
| HanXRQChr08g0213821 | glycoside hydrolase family 31 - GH31 (alpha-xylosidase) | - |
| HanXRQChr14g0461441 | | glycoside hydrolase family 32 - GH32 | | --- | | - |
| HanXRQChr04g0116041 | homologous to *A. thaliana* PMR5 (Powdery Mildew Resistant) | - |
| HanXRQChr17g0558861 | lectin (PAN-2 domain) | - |
| HanXRQChr09g0267051 | lipase acylhydrolase (GDSL family) | - |
| HanXRQChr02g0047121 | mannose-binding lectin | - |
| HanXRQChr09g0238641 | mannose-binding lectin | - |
| HanXRQChr13g0398251 | peroxiredoxin | - |
| HanXRQChr03g0072241 | Ser carboxypeptidase (Peptidase family S10) | - |
