# Supplementary figures and images for: Extracellular vesicles: a missing component in plant cell wall remodeling
Source: J Exp Bot. 2018 Jul 11;69(20):4655–8. doi: 10.1093/jxb/ery255 (PMC6137967; doi:10.1093/jxb/ery255)

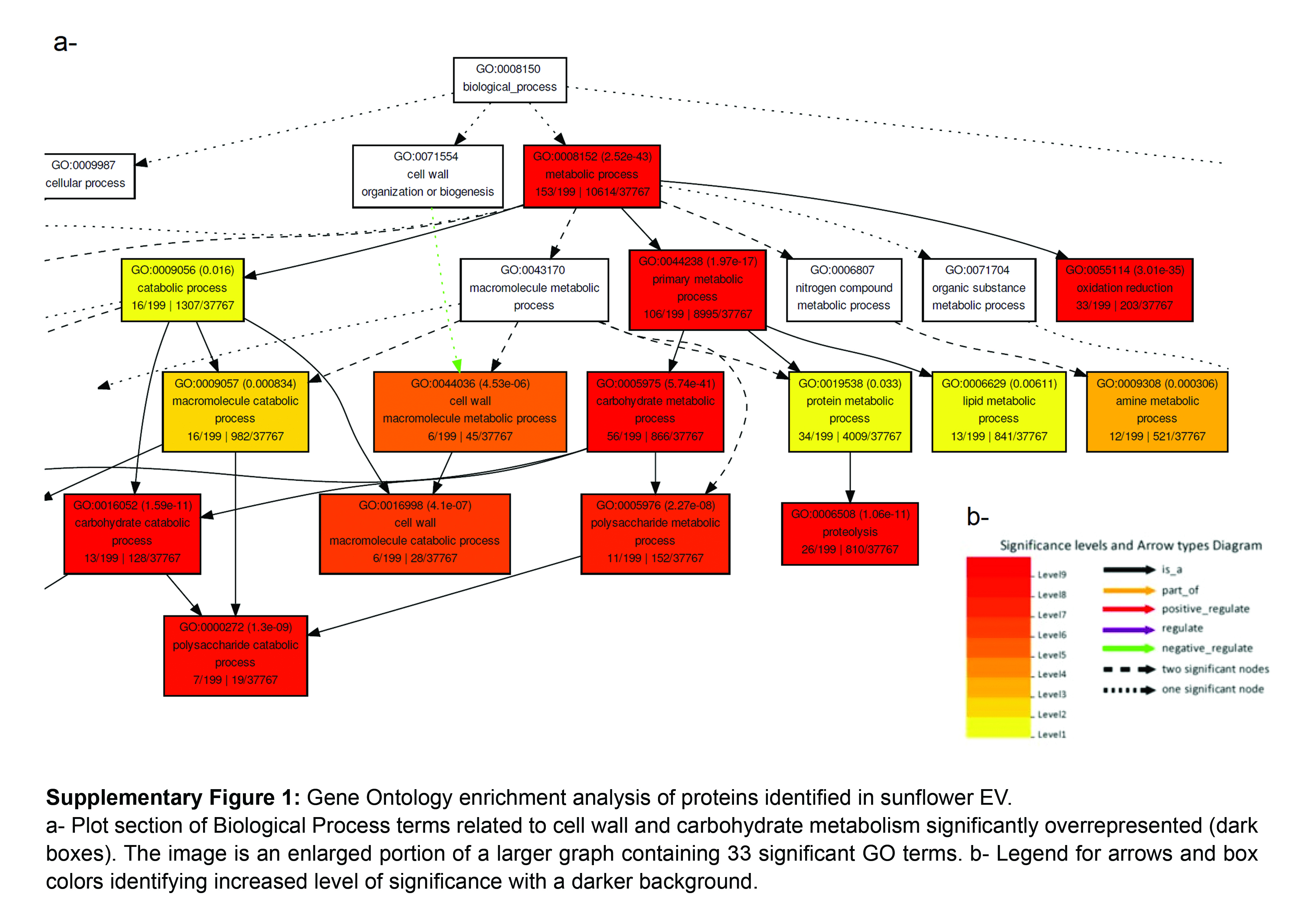

Supplement: Supplementary Figure S1 [file ery255_suppl_supplementary_figure_s1.jpeg]
